# Structural insights into the activation of MST3 by MO25

**DOI:** 10.1016/j.bbrc.2012.12.113

**Published:** 2013-02-15

**Authors:** Youcef Mehellou, Dario R. Alessi, Thomas J. Macartney, Marta Szklarz, Stefan Knapp, Jonathan M. Elkins

**Affiliations:** a MRC Protein Phosphorylation Unit, College of Life Sciences, University of Dundee, Dow Street, Dundee, DD1 5EH Scotland, UK; b Structural Genomics Consortium, Nuffield Department of Clinical Medicine, University of Oxford, Old Road Campus Research Building, Roosevelt Drive, Oxford OX3 7DQ, UK

**Keywords:** LB, Luria–Bertani, MST, mammalian sterile 20 (Ste20)–like kinase, MO25, mouse protein-25, PEI, polyethyleneimine, STRAD, STE20-related adapter protein, YSK1, yeast SPS/STE20-related kinase-1, Protein kinase, LKB1, Signal transduction, Protein structure and STE20

## Abstract

The MO25 scaffolding protein operates as critical regulator of a number of STE20 family protein kinases (e.g. MST and SPAK isoforms) as well as pseudokinases (e.g. STRAD isoforms that play a critical role in activating the LKB1 tumour suppressor). To better understand how MO25 interacts and stimulates the activity of STE20 protein kinases, we determined the crystal structure of MST3 catalytic domain (residues 19–289) in complex with full length MO25β. The structure reveals an intricate web of interactions between MST3 and MO25β that function to stabilise the kinase domain in a closed, active, conformation even in the absence of ATP or an ATP-mimetic inhibitor. The binding mode of MO25β is reminiscent of the mechanism by which MO25α interacts with the pseudokinase STRADα. In particular we identified interface residues Tyr223 of MO25β and Glu58 and Ile71 of MST3 that when mutated prevent activation of MST3 by MO25β. These data provide molecular understanding of the mechanism by which MO25 isoforms regulates the activity of STE20 family protein kinases.

## Highlights

► Elucidation of the structure of the catalytic core of MST3 complexed with its MO25β regulatory subunit. ► Define key conserved interface residues on MO25β and MST3 that interact with one another. ► First structure of MO25β isoform to be reported. ► Findings provide greater insight into how MO25 isoforms can function as master regulators of STE20 kinase.

## 1 Introduction

Mouse Protein-25 (MO25) is a highly conserved scaffolding protein originally discovered as a highly evolutionary conserved protein expressed at the early cleavage stage of mouse embryogenesis [1]. Mammals possess two closely related isoforms termed MO25α and MO25β, which share 79% sequence identity [2]. One of the most investigated roles of MO25α is as one of the core components of the LKB1 tumour suppressor complex that regulates proliferation, metabolism and polarity by phosphorylating and stimulating 14 AMPK family protein kinases [3]. The LKB1 kinase complex is a heterotrimer consisting of the protein kinase LKB bound to a catalytically inactive pseudokinase termed STRAD (of which there are two isoforms termed STRADα and STRADβ) and MO25 [4–6]. Association of LKB1 with MO25 and STRAD stimulates LKB1 protein kinase activity [2,4] and is essential for activation of AMPK family kinases [7,8].

The crystal structure of monomeric MO25α revealed that it consists of seven helical repeats arranged in a distinctive horseshoe shape distantly related to the Armadillo proteins with a concave surface aligned with highly conserved residues and a less conserved convex surface fold [9]. Subsequent work analysing the structure of the heterodimeric STRAD:MO25 [10] and the heterotrimeric LKB1:STRAD:MO25 [11] complexes showed that the concave surface of MO25 formed an intricate network of interactions with the αC-helix of STRADα, reminiscent of the mechanism by which CDK2 interacts with cyclin A. MO25α acts to stabilise STRADα in an active conformation with an ordered T-loop that binds ATP and hence capable of binding to and activating LKB1 [10,11]. Activation of LKB1 is prevented by mutations that are predicted to destabilise the active conformation of STRADα namely those that prevent binding of STRADα to MO25α and ATP [11]. MO25α also stimulates LKB1 catalytic activity by binding directly with the T-loop of LKB1 and stabilises it in an active conformation [11].

Recent work has demonstrated that MO25 isoforms also interact with at least five distinct protein kinases that are related to STRADα/STRADβ and belong to the STE20 branch of mammalian kinases. These include SPAK and OSR1 that are important regulators of ion homeostasis and blood pressure [12] as well as MST3, MST4, YSK1 that control morphogenesis and polarity [13,14]. The catalytic activity of SPAK and OSR1 is stimulated over 100-fold by binding to MO25 isoforms, whereas MST3, MST4 and YSK1 are activated three- to four-fold. To learn more about the roles of MO25 in regulating STE20 protein kinases we have co-crystallised the catalytically active kinase domain of MST3 with MO25β. The structure reveals the intricate mechanism by which MO25β binds to MST3 and enables the construction of mutants that prevent activation of MST3 by MO25β.

## 2 Materials and methods

### 2.1 Reagents

Tissue-culture reagents were from Life Technologies. P81 phospho-cellulose paper was from Whatman and [γ-^32^P]-ATP was from Perkin Elmer. Myelin basic protein was from Sigma.

### 2.2 Expression and purification of MST3 and MO25β complex for crystallisation

MST3 and MO25β clones were transformed into *E. coli* BL21 (DE3) competent cells containing the pRARE2 plasmid from the commercial Rosetta strain, and the transformants were used to inoculate 50 ml of Luria–Bertani (LB) medium containing 50 μg/ml kanamycin (MST3) or 100 μg/ml ampicillin (MO25β) with the addition of 34 μg/ml chloramphenicol. These cultures were incubated overnight at 37 °C. For each protein, 3× 10 ml of the overnight culture was used to inoculate 3× 1 l of LB medium with 40 μg/ml kanamycin or 80 μg/ml ampicillin and grown at 37 °C until an OD_600_ of 0.4–0.5 was reached. The temperature was then reduced to 20 °C. When the OD600 reached 0.6, expression was induced by addition of 0.5 mM IPTG. Expression was continued overnight. The cells were harvested by centrifugation. The MST3 cells were resuspended in Binding Buffer. The MO25β cells were resuspended in Buffer 1. The resuspended cells were frozen until further use.

For purification the cells were thawed and lysed by sonication on ice. PEI (polyethyleneimine) was added to a final concentration of 0.15% and the cell debris and precipitated DNA were spun down. MST3 was purified by passing the supernatant through a gravity column of 5 ml Ni-Sepharose resin (GE Healthcare). The resin was washed with 50 ml of Binding Buffer containing 1 M NaCl and 40 mM imidazole, 50 ml of Binding Buffer containing 60 mM imidazole before the protein was eluted with 25 ml of Binding Buffer containing 250 mM imidazole. The eluted protein was further purified by gel filtration chromatography using an S200 16/60 column (GE Healthcare) in 20 mM Tris pH 7.5, 200 mM NaCl, 0.5 mM TCEP. MO25β was purified by passing the supernatant through a gravity column of 5 ml Glutathione-Sepharose (GE Healthcare). The resin was washed with 6x 10 ml of Buffer 1 and 6x 10 ml of Buffer 2. Prescission protease was added to the resin and incubated overnight. The MO25β protein was then eluted with 50 ml of Buffer 2.

The MST3 protein was concentrated to 2.4 mg/ml and the MO25β protein was concentrated to 7.8 mg/ml. The concentrated proteins were mixed in an equimolar ratio and injected onto an S200 16/60 gel filtration column pre-equilibrated in 20 mM Tris pH 7.5, 200 mM NaCl, 0.5 mM TCEP. Fractions containing the protein complex were pooled and concentrated to 9.7 mg/ml. Protein identities were confirmed by mass spectrometry under denaturing conditions (MST3: expected 33257 Da, observed 33257 Da; MO25β: expected 39225 Da, observed 39225).

### 2.3 Structure determination

Crystals were obtained using the sitting drop vapour diffusion method at 4 °C. The protein (9.7 mg/ml) was mixed 1:1 with a reservoir solution containing 0.2 M Na/KPO4, 10% PEG 3350, 10% Ethylene Glycol in a drop size of 150 nl. Crystals were cryoprotected by transfer into reservoir solution containing 25% ethylene glycol. Crystals were then flash-frozen in liquid nitrogen and diffraction data were collected at Diamond beamline I04-1. Data collection statistics can be found in Table 1.

The diffraction data was indexed and integrated using MOSFLM [15] and scaled using SCALA [16]. The structure was solved by molecular replacement using PHASER [17]. The search models used were PDB IDs 3CKW (human MST3) and an ensemble of 3GNI, 1UPK and 2WTK (MO25). Two molecules of the MST3:MO25β complex were present in the asymmetric unit. The model was built in Coot [18] and refined with REFMAC5 [19]. Tight NCS restraints were applied during the refinement, which included refinement of TLS parameters.

Further details on general methods and plasmids; Buffers, cell culture, transfection and immunoprecipitation, expression MST3 and kinase activity measurements, expression and purification of MO25 mutants in *E. coli* is provided in the supplementary section.

## 3 Results and discussion

### 3.1 Description of the MST3:MO25 complex

We crystallised a complex of an MST3 catalytic fragment (residues 19–289) with full length MO25β. The MST3 numbering is given according to that of isoform b (NCBI NP_001027467.2) (Table 1). The 19–289 fragment encompasses the kinase domain, but lacks the C-terminal non-catalytic domain that contains a WIF motif (residues 325–327 in MST3). The equivalent residues in STRADα (WEF: residues 429-431) bind to a C-terminal hydrophobic pocket on MO25α [9,20].

**Fig. 1:**
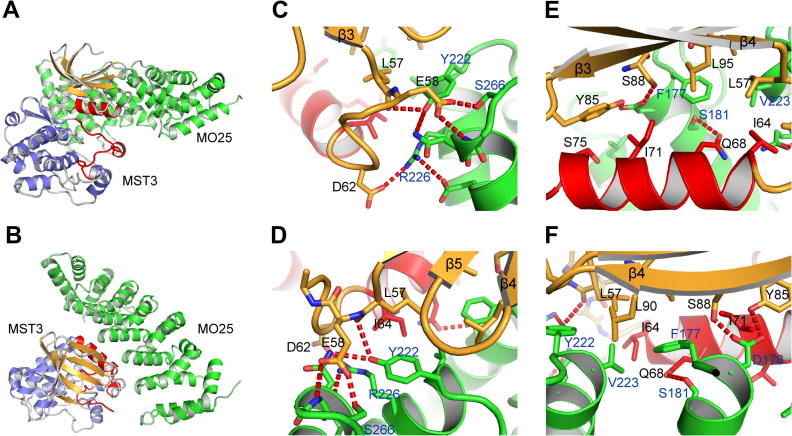
Structure of the MST3:MO25β complex. (A) Structural overview with MO25β coloured green, the N-terminal lobe of MST3 coloured brown and the C-terminal lobe coloured blue. The activation loop and helix αC are coloured red. (B) As (A) with an approximately 90° rotation. (C, D) Two views of the hydrogen bonding network that links the loop between strand β3 and αC with MO25β. (E and F) Two views of the interactions between MST3 and MO25β involving αC and β3 of MST3. (For interpretation of the references to colour in this figure legend, the reader is referred to the web version of this article.)

**Fig. 2:**
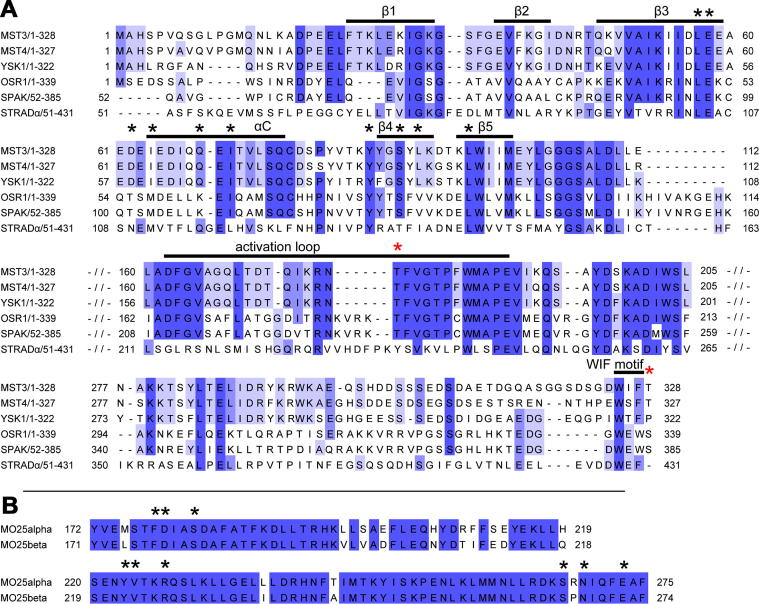
Sequence alignments of (A) Relevant regions of kinases activated by MO25, and (B) MO25 isoforms. Residues of MST3 or MO25β that are on the interface between MST3 and MO25β are marked with a black ∗ above the alignment. Known phosphorylation sites of MST3 are marked with a red ∗. Full sequence alignments can be seen in the Supplementary material. (For interpretation of the references to colour in this figure legend, the reader is referred to the web version of this article.)

Two copies of the MST3:MO25β complex occupied the crystallographic asymmetric unit and in each case the same binding arrangement for MST3 and MO25β was observed. The domain arrangement resembles that of the previously published complex between STRADα and MO25α [10]. MO25β binds to MST3 adjacent to the helix αC which has a key role regulating kinase activity in most kinases. Structural comparison with the STRADα:MO25α complex revealed additional interactions involving MST3 strands β4 and β5 (Fig. 1). These interactions primarily involve three out of the six structural repeats of MO25β. The residues of MO25β that interact with MST3 are highly conserved in both MO25 isoforms (Fig. 2B).

The extensive number of interactions between MST3 and MO25β can be divided into three sections: (a) Hydrogen bonding interactions involving the central part of αC and strand β4. There are hydrogen bonds from the side-chains of Tyr85 and Ser88 to Asp178 of MO25β, as well as between the side-chains of Gln68 and Ser181 of MO25β (Fig. 1E and F). (b) Central hydrophobic interactions involving the N-terminal part of αC and residues from strands β3, β4 and β5. Leu57, Ile64, Leu90, Leu95 from MST3 form this core hydrophobic patch with Phe177 and Val223 from MO25α (Fig. 1E and F). (c) A network of hydrogen bonding interactions involving the loop between MST3 strand β3 and αC. Glu58 is at the centre of this network, forming hydrogen bonds to the backbone nitrogen atom of MO25α Asn268 and the side-chain of MO25α Ser266. Additional hydrogen bonds are formed between the side-chains of Asp62 and MO25α Arg226, and between the backbone oxygen of Leu57 and MO25β Tyr222 (Fig. 1C and D).

These results suggest that MO25β activates MST3 by stabilisation of αC in an active position. In inactive kinases it is often possible for αC to assume an outward position, displaced away from the ATP binding site. As MO25β binds to αC and also to β4 and β5 it fixes the position of αC relative to β4, β5 and the rest of the N-terminal lobe of the kinase domain. Therefore the binding energy of MO25β to MST3 is used to pre-organise the catalytically important regions of the kinase domain, a mechanism frequently observed for activators of kinases. The stabilisation of αC also leads to the partial stabilisation of the activation loop via a number of interactions between them, in particular in the region around the DFG motif. However, MST3 will be activated further by auto-phosphorylation on Thr178.

**Fig. 3:**
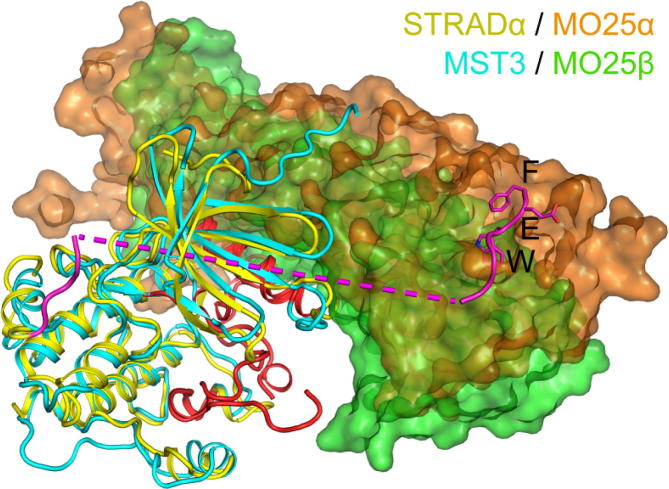
Superimposition of MST3:MO25β structure with STRADα:MO25α. MST3 is shown in cyan, and STRADα in yellow with its C-terminal region including WEF motif in magenta. MO25β bound to MST3 is shown as a green surface, and MO25α bound to STRADα is shown as a brown surface. (For interpretation of the references to colour in this figure legend, the reader is referred to the web version of this article.)

The binding angle between the MST3 kinase domain and MO25β is slightly different to that between STRADα and MO25α (Fig 3). This difference may be however influenced by differences in crystal packing environment, but it might also be a consequence of the lack of a WEF peptide binding to MO25β. The difference in binding angle does not significantly change the surface area of interaction, or the angle of the key kinase regulatory element helix αC.

### 3.2 Mutational analysis of interface residues

**Fig. 4:**
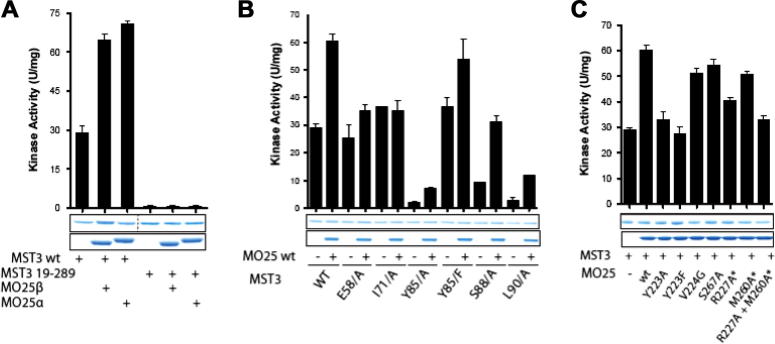
Mutational analysis of how truncation of non-catalytic WIF-motif of MST3 and interface residues impacts on basal kinase activity and activation by MO25 isoforms. (A and B) The indicated wild type and mutant forms of MST3 were purified from HEK293 cells and were assayed in the presence or absence of ten-fold excess of wild type full length MO25α or MO25β, purified from *E. coli*. The activity in the presence of MO25α is reported relative to the activity measured in the absence of MO25α. Assays were undertaken in triplicate and data presented as mean ± s.d. Coomassie gels are shown to illustrate the relative levels of wild type and mutant. (C) as in (A) except that the wild-type or indicated mutants of MO25α were employed.

To assess the importance of the interface residues in mediating the activation of full length MST3 by MO25, we compared basal and MO25-stimulated activity of full length MST3 and the crystallised MST3 (19–289) fragment. The data showed that the full length MST3 was activated by both MO25α and MO25β isoforms as previously reported [20], whilst the crystallised MST3 (19–289) fragment was devoid of catalytic activity and the addition of either MO25α or β did not result in any further activation. A fragment of MST3 that encompasses the WIF motif (i.e. residues 19–319) lacking in the crystallised MST3 displayed similar basal and MO25 induced activity to full length MST3 (Fig. 4A).

The crystal structure of MST3 (19–289) in complex with MO25β was used to design mutations in full length MST3 as well as MO25 isoforms to insight into the residues needed for binding between the two proteins, which result in an increased kinase activity of MST3 by MO25. We first mutated residues on full length MST3 that were implicated to interact with MO25β (Glu58, Ile71, Tyr85, Ser88, and Leu90) and tested how this impacted on the basal MST3 activity and its activation by MO25α. This revealed that mutation of Glu58 or Ile71 to Ala did not affect basal activity but prevented MST3 activation by MO25α. Mutation of Tyr85 to Ala markedly inhibited basal MST3 activity, while mutating Tyr85 to Phe maintained basal activity and partially supressed activation by MO25α. Mutation of Ser88 and Leu90 markedly reduced basal MST3 activity but the activity of these mutants was still enhanced 2- to 3-fold by MO25α (Fig. 4B). We also mutated a number of residues on MO25α implicated in binding to MST3. This revealed that mutation of MO25α Tyr223 that interacts with MST3 Leu57 abolished activation of MST3 by MO25α, similarly to the previously reported R227A + M260A double mutant [20] (Fig. 4C).

Three of these mutations reduced basal MST3 activity significantly. The aromatic ring of Tyr85 is probably important for stabilisation and binding of strands β4 and β5 against αC, since Y85F retained basal activity compared to Y85A, which reduced basal activity (Fig. 4B). In the case of S88A and L90A, which also reduced basal activity, the same explanation would apply: secure binding of β4 to β5 is presumably important for the stability of the N-terminal lobe. The prevention of MO25 activation by the I71A and E58A mutants shows that, respectively, the central hydrophobic binding interface and the interactions between the β3–αC loop and MO25 are both essential for activation by MO25. The essential nature of the β3–αC loop interactions with MO25 are emphasised by the inability of MO25α Y223A or Y223F to activate MST3.

### 3.3 Comparison of MST3 activation to OSR1 and SPAK

The kinases OSR1 and SPAK are activated around 25 times more strongly by MO25 than are MST3, MST4 and YSK1. Analysis of the MST3 residues at the interface with MO25β reveals that many of the interacting residues are conserved across all five of these kinases (Fig. 2A). Of the five MST3 residues on β4 and β5, four are completely conserved (Tyr85, Ser88, Leu95) while Leu90 is conservatively substituted by Val in OSR1 and SPAK. Of the six MST3 residues on β3 and αC that interact with MO25β, three are completely conserved (Leu57, Glu58 and Ile71) and two are conservatively substituted in OSR1 and SPAK (Asp62 (as Thr) and Ile64 (as Met)). However, one key difference is Gln68 which is replaced by Leu in OSR1 and SPAK. Gln68 provides a polar side-chain at the centre of an otherwise hydrophobic patch (Fig. 1E and F). Replacement of Gln68 with Leu would significantly increase the interacting surface that stabilizes this hydrophobic interaction. This would presumably increase the affinity of MO25 for OSR1 and SPAK and therefore provide more binding energy for stabilisation of a catalytically active kinase conformation.

There is one other potentially key difference between MST3, MST4 or YSK1, and OSR1 or SPAK, which is that the former all have a WXF motif, while the latter have a WEW motif. STRADα also has a WXF motif like MST3, and the structure of STRADα with MO25α [10] suggests that replacement of WXF with WXW may increase affinity by increasing the hydrophobic interaction area between the WXF motif and MO25α, and possibly providing an additional hydrogen bond to Asn261 of MO25α.

### 3.4 The C-terminal non-catalytic domain of MST3 is essential for activity

Our results show that the C-terminal non-catalytic region of MST3 is essential for activity, even in the absence of MO25. However, mutation of the Trp and Phe residues in the WIF motif to Ala in full length MST3 or MST4 only reduced basal kinase activity 2- to 3-fold and activity of these mutants was still stimulated by addition of MO25α [20]. Moreover, mutation of the equivalent residues in the WIF motif of YSK1 barely affected basal or MO25 stimulated activity [20]. Thus residues other than the WIF motif may also account for lack of activity of crystallised forms of MST isoforms. The published structures of MST3 with a variety of co-factors (ADP, adenine, ADP + Mn) [21], as well as other unpublished MST3 structures in the PDB (3CKW, 3CKX), were all also derived from MST3 constructs lacking the C-terminal WIF motif. The structure of YSK1 (PDB ID 2XIK) was also derived from a construct lacking a C-terminal WIF motif, as were the structures of MST4 [22]. Further work is therefore warranted to elucidate the mechanism by which the non-catalytic domain regulates kinase activity of these enzymes.

The structures of STRADα with MO25α [10,11] showed that the C-terminal region including the WEF motif links the C-terminal lobe of the STRADα kinase domain to the binding site on MO25α located above the N-terminal lobe of STRADα. C-terminal extensions to kinase domains that bind to the N-terminal lobe and activate the kinase are a feature of many kinases, in particular those of the AGC family. For STRADα, and MST3, to make this connection in an intramolecular way requires that this C-terminal region crosses the front of the ATP binding site or the substrate-binding region. Therefore in addition to stabilisation of the kinase domain by linking the C-terminal lobe to the N-terminal lobe via MO25, there may also be direct effects on interactions at the active site. In the absence of MO25 the C-terminal extension to MST3 may also exert its activating effect by interaction with these important regions, or perhaps by binding of the WIF motif to a location elsewhere on the kinase domain.

### 3.5 Relevance to activation of MST3 substrates

MST3 has been reported to activate NDR2 protein kinase [23] by phosphorylating the Thr of its hydrophobic motif (sequence FLNYTY). Recently it has been reported that in addition to autophosphorylating on Thr178 of its activation loop, MST3 also autophosphorylates on Thr328 which is located just after the WIF motif (sequence WIFTI) [24]. Clearly these sequence motifs have a strong resemblance to each other, being of the form Φx_(1−2)_ΦTΦ where Φ is a hydrophobic residue. It may be that in the mechanism of NDR2 phosphorylation an exchange takes place whereby the (phosphorylated) MST3 WIF motif binds to NDR2 in place of its hydrophobic motif, allowing the NDR2 hydrophobic motif to bind to the substrate site on MST3. A recent paper has shown that in the fungi *Neurospora crassa* the HYM1 (MO25) protein regulates COT1 (NDR), acting to scaffold the NDR kinase with its activating kinase POD6 [25]. In fission yeast, it was reported that the equivalent protein Pmo25 (MO25) and the kinase Nak1 were required for activation of the kinase Orb6 (NDR) [26]. Orb6 has 50% sequence identity to human NDR2 and has an equivalent hydrophobic motif FLGYTY, while Pmo25 is 51%/52% identical to MO25α/MO25β and Nak1 is 51% identical to human MST3 (its closest relation) and also possesses a WEF motif followed by a potential phospho-acceptor (sequence WEFGT, amino acids 321–325). It therefore may be that human MO25 would have an activating function for MST3 on NDR, in an evolutionarily conserved mechanism.

## Author contributions

JME and MS undertook structural studies (Fig. 1), YM undertook functional analysis (Fig. 3) JME, YM, DRA and SK planned the experiments, analysed the experimental data and wrote the manuscript.

## Figures and Tables

**Table 1:** Data collection and refinement statistics.

PDB ID	3ZHP
Space group	*P*2_1_
No. of molecules in the asymmetric unit	2
Unit cell dimensions *a*, *b*, *c* (Å) *α*, *β*, *γ* (°)	64.8, 120.3, 98.9 90.0, 99.7, 90.0

*Data collection*	
Beamline	Diamond I04-1
Resolution range (Å)^a^	63.88–2.90 (3.06–2.90)
Unique observations^a^	32,108 (4687)
Average multiplicity^a^	4.3 (4.3)
Completeness (%)^a^	96.8 (97.3)
*R*_merge_^a^	0.07 (0.78)
Mean ((*I*)/σ(*I*))^a^	10.0 (1.7)

*Refinement*	
Resolution range (Å)	63.96–2.90
*R*-value, *R*_free_	0.23, 0.26
r.m.s. deviation from ideal bond length (Å)	0.009
r.m.s. deviation from ideal bond angle (°)	0.99
Ramachandran outliers Most favoured	0.61% 95.57%

a Values within parentheses refer to the highest resolution shell.
