# Supplementary material for: Structural insights into the activation of MST3 by MO25
Source: Biochem Biophys Res Commun. 2013 Feb 15;431(3):604–9. doi: 10.1016/j.bbrc.2012.12.113 (PMC3725419; doi:10.1016/j.bbrc.2012.12.113)
Supplement: Supplementary data — Supplementary material [file mmc1.docx]

# Structural Insights into the Activation of MST3 by MO25

Youcef Mehellou^1^, Dario R Alessi^1^, Thomas J Macartney^1^, Marta Szklarz^2^, Stefan Knapp^2^ and Jonathan M. Elkins^2^

^1^ MRC Protein Phosphorylation Unit, College of Life Sciences, University of Dundee, Dow Street, Dundee DD1 5EH, Scotland.

^2^ Structural Genomics Consortium, Nuffield Department of Clinical Medicine, University of Oxford, Old Road Campus Research Building, Roosevelt Drive, Oxford, OX3 7DQ, U.K.

## Supplementary Section

### Sequence alignment of kinases activated by MO25


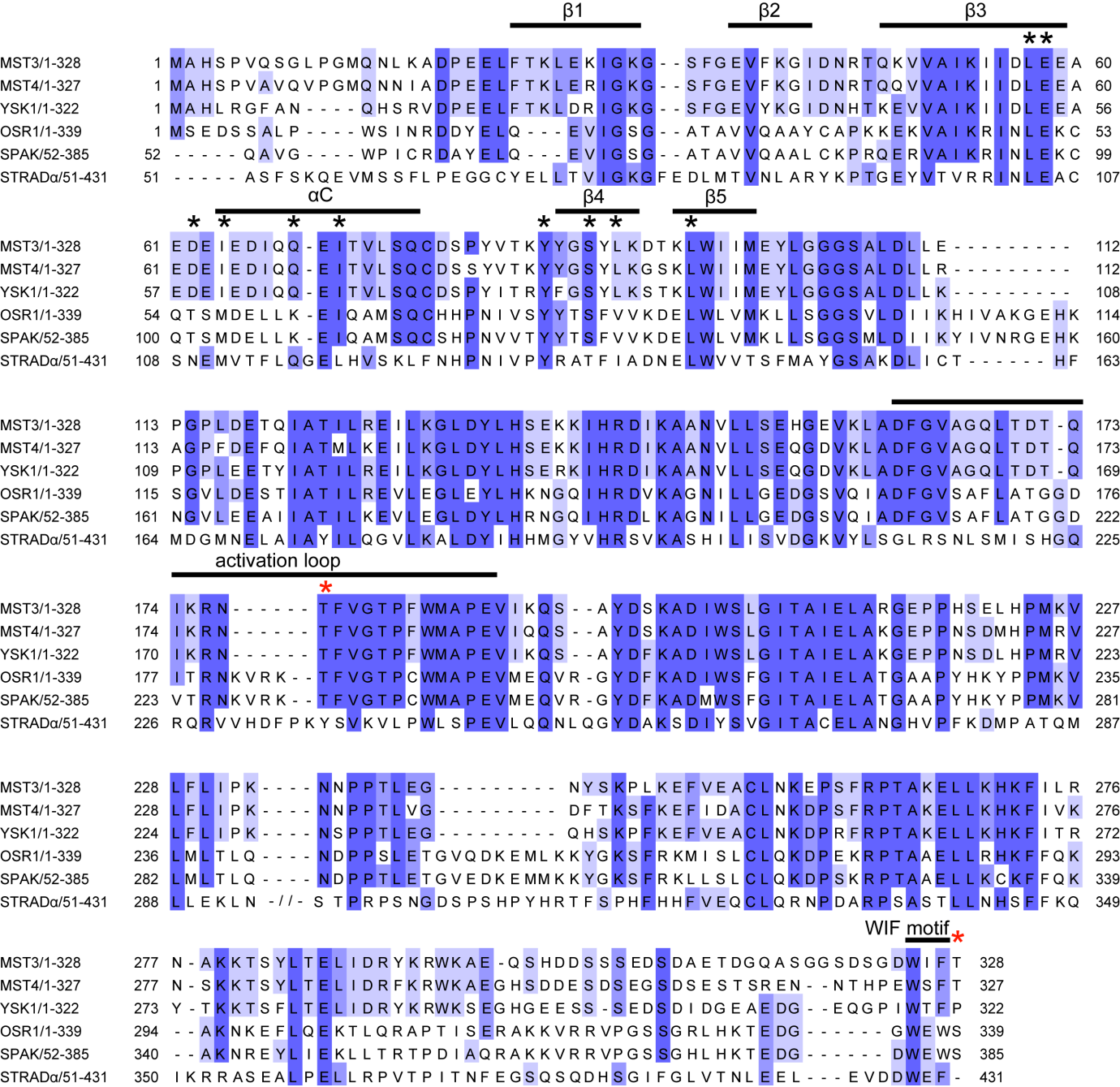


Residues of MST3 that are on the interface between MST3 and MO25β are marked with a black * above the alignment. Known phosphorylation sites of MST3 are marked with a red *.

### Sequence alignment of human MO25 isoforms


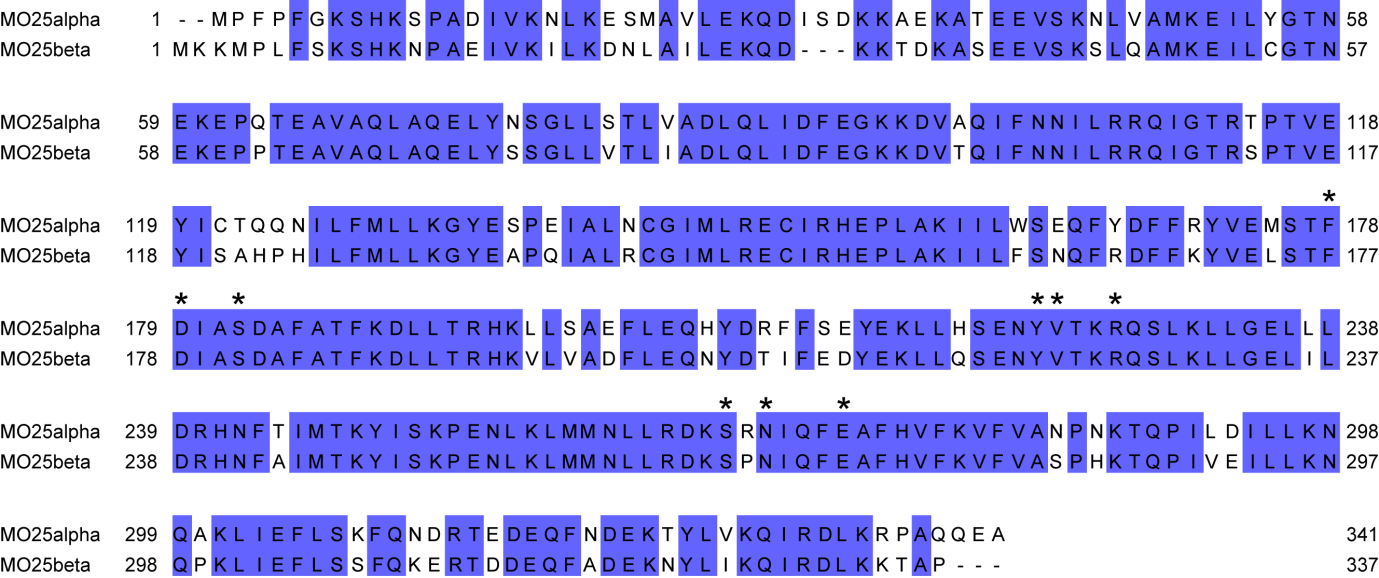


Residues of MO25β that are on the interface between MST3 and MO25β are marked with a black * above the alignment.

### General methods and plasmids

DNA for MST3 residues 19-289 (also known as STK24, NCBI NP_001027467.2) was PCR amplified and subcloned into an in-house pET-based vector carrying kanamycin resistance, pNIC28-Bsa4, using ligation-independent cloning. The resulting plasmid expressed the kinase domain of MST3 with an N-terminal hexahistidine tag and TEV (tobacco etch virus) protease tag cleavage site (extension MHHHHHHSSGVDLGTENLYFQ*SM-). MO25β (CAB39L, NM_030925.2) was subcloned as a BamHI/NotI into pGEX6P-1 for bacterial expression as an N-terminally tagged GST-fusion protein containing a Precission protease cleavage site to enable GST removal following glutathione-agarose purification.

### Buffers

HEK 293 cells lysis buffer: 50 mM Tris-HCl (pH 7.5), 1 mM EGTA, 1 mM EDTA, 1% (w/v) Nonidet P40, 1 mM sodium orthovanadate, 50 mM sodium fluoride, 5 mM sodium pyrophosphate, 0.27 M sucrose, 0.1% (v/v) 2-mercaptoethanol, 1 mM benzamidine, and 0.1 mM PMSF. Bacterial lysis buffer: 50 mM Tris-HCl (pH 7.8), 150 mM NaCl, 0.27 M sucrose, 1 mM benzamidine, 1 mM EGTA, 1 mM EDTA, 0.1 mM PMSF, and 0.01% (v/v) β- mercaptoethanol, supplemented with 0.5 mg/ml lysozyme and 0.3 mg/ml DNAse-I. Buffer A: 50 mM Tris-HCl (pH 7.5), 0.1 mM EGTA and 0.1% (v/v) 2- mercaptoethanol. Binding buffer: 50 mM HEPES pH 7.4, 500 mM NaCl, 5% glycerol, 20 mM imidazole, 0.5 mM TCEP, 0.2 mM PMSF. Buffer 1: 50 mM Tris-HCl pH 8.0, 1 mM EDTA, 1 mM EGTA, 1 mM sodium orthovanadate, 25 mM NaF, 5 mM DTT and 1:2000 dilution of Protease Inhibitor Cocktail (Sigma). Buffer 2: 50 mM Tris-HCl pH 8.0, 200 mM NaCl, 0.1 mM EGTA, 5 mM DTT. SDS sample buffer: contains 50 mM Tris-HCl (pH 6.8), 1% (w/v) SDS, 10% (v/v) glycerol, 0.005% (w/v) bromophenol blue, and 1% (v/v) 2-mercaptoethanol. TBS-T buffer: 50 mM Tris-HCl (pH 7.5), 0.15 M NaCl, and 0.25% (v/v) Tween.

### Cell culture, transfection and immunoprecipitation

HEK 293 cells were cultured on 10 cm diameter dishes in 10 ml of DMEM supplemented with 10% (v/v) fetal bovine serum, 2 mM L-glutamine, 100 U/ml penicillin, and 0.1mg/ml streptomycin. For transfection, 3 µg of the pEBG-2T DNA constructs encoding N-terminal GST or FLAG- fusions of wild type and mutant MST3 kinase was mixed with 20 µl of 1 mg/ml polyethylamine (polysciences) in 1 ml of plain DMEM for each dish. The mixture was left to stand for 20 minutes at room temperature and added onto the cells. Cells were lysed 36 h post-transfection, and the clarified lysates were incubated for 1 h on a rotating platform with glutathione-Sepharose (GE Healthcare; 10 µl/dish of lysate) or anti-FLAG M2 beads (Sigma, A2220, 10 µl/dish of lysate), which had been previously equilibrated in lysis buffer. The beads were washed once with lysis buffer containing 0.15 M NaCl, three times with lysis buffer containing 0.5 M NaCl and twice with buffer A. GST-tagged proteins were eluted from the resin by incubation with the same buffer A containing 0.27 M sucrose and 20 mM of reduced glutathione (pH 7.5), while FLAG-tagged proteins were eluted off the beads using FLAG peptide (DYKDDDDK, DSTT; EP3840) at a centration of 0.2 mg/ml. The beads were then removed by filtration through a 0.44 µm filter, and the eluted protein aliquoted and stored at -80°C.

### Expression MST3 and kinase activity measurements.

The activity of recombinant MST3 kinases was assayed using myelin basic protein (MBP) as a substrate at a concentration of 0.3 mg/ml. Phosphotransferase activity of each kinase was measured in a total assay volume of 50 µl consisting of 50 mM Tris-HCl (pH 7.5), 0.1 mM EGTA, 0.1% (v/v) 2-mercaptoethanol, 10 mM magnesium acetate, 0.1 mM [γ-32P]ATP (200 cpm/pmol). MST3 kinases were used at 0.1 µM while MO25 was used at a concentration of 1 µM as reported [1]. The assays were carried out at 30 °C and were terminated after 45 minutes by applying 40 μl of the reaction mixture onto P81 membranes. These were washed in phosphoric acid, and the incorporated radioactivity was measured by scintillation counting as described previously for MAP kinase [2]. One unit (U) of activity represents the incorporation to the substrate of 1 nmol of γ-32P per minute.

### Expression and purification of MO25 mutants in E. coli

Cells transformed with the appropriate expression plasmid were grown in Luria-Bertani medium to A600 = 0.7 at 37 °C. Protein expression was induced by the addition of 0.25 mM isopropyl β-D-1-thiogalactopyranoside (IPTG) and cells incubated for a further 16 h at 26 °C. Cells were harvested by centrifugation for 30 min at 3,500 g and resuspended in ice-cold bacteria lysis buffer. Cells were lysed by sonication (10 × 10 s pulses) and the lysates underwent centrifugation at 26,000 g to remove residual debris before passing the supernatant through a 0.44 μm filter. The cleared lysates were subsequently incubated for 1 h on a rotating platform with glutathione-Sepharose (1 ml/l of E. coli culture) pre-equilibrated in bacterial lysis buffer. The beads were then washed with 10 CV with bacterial lysis buffer lacking DNase-1 and lysozyme and a further 10 times the volume of the beads with high-salt lysis buffer containing 0.5 M NaCl and lacking DNAsse-1 and lysozyme. The beads were re-equilibrated in 10 times the volume of the beads with buffer A, and the proteins were eluted by incubation with PreScission protease (15 µg/mg of protein) at room temperature for 30 min and subsequently at 4 °C for 16 h. The beads were then washed with 10 times the beads volume with buffer A to elute the protein, and this supernatant was then passed back over a 1-ml column of glutathione-Sepharose equilibrated in buffer A to remove GST contaminants. The supernatant of MO25 mutants were concentrated at this stage, run on an SDS-PAGE gel to check purity and subsequently used in the kinase assays.

### References

[1] B.M. Filippi, P. de Los Heros, Y. Mehellou, I. Navratilova, R. Gourlay, M. Deak, L. Plater, R. Toth, E. Zeqiraj, D.R. Alessi, MO25 is a master regulator of SPAK/OSR1 and MST3/MST4/YSK1 protein kinases, EMBO J 30 (2011) 1730-1741.

[2] D.R. Alessi, P. Cohen, A. Ashworth, S. Cowley, S.J. Leevers, C.J. Marshall, Assay and expression of mitogen-activated protein kinase, MAP kinase kinase, and Raf, Methods Enzymol 255 (1995) 279-290.
